# *Pseudomonas aeruginosa* Ld-08 isolated from *Lilium davidii* exhibits antifungal and growth-promoting properties

**DOI:** 10.1371/journal.pone.0269640

**Published:** 2022-06-17

**Authors:** Mohammad Sayyar Khan, Junlian Gao, Mingfang Zhang, Jing Xue, Xiuhai Zhang

**Affiliations:** 1 Microbiology Division, Institute of Biotechnology and Genetic Engineering (IBGE), The University of Agriculture, Peshawar, Khyber Pakhtunkhwa, Pakistan; 2 Beijing Academy of Agriculture and Forestry Sciences, Beijing, China; COMSATS University Islamabad - Abbottabad Campus, PAKISTAN

## Abstract

A plant growth-promoting and antifungal endophytic bacteria designated as Ld-08 isolated from the bulbs of *Lilium davidii* was identified as *Pseudomonas aeruginosa* based on phenotypic, microscopic, and 16S rRNA gene sequence analysis. Ld-08 exhibited antifungal effects against *Fusarium oxysporum*, *Botrytis cinerea*, *Botryosphaeria dothidea*, and *Fusarium fujikuroi*. Ld-08 showed the highest growth inhibition, i.e., 83.82±4.76% against *B*. *dothidea* followed by 74.12±3.87%, 67.56±3.35%, and 63.67±3.39% against *F*. *fujikuroi*, *B*. *cinerea*, and *F*. *oxysporum*, respectively. The ethyl acetate fraction of Ld-08 revealed the presence of several bioactive secondary metabolites. Prominent compounds were quinolones; 3,9-dimethoxypterocarpan; cascaroside B; dehydroabietylamine; epiandrosterone; nocodazole; oxolinic acid; pyochelin; rhodotulic acid; 9,12-octadecadienoic acid; di-peptides; tri-peptides; ursodiol, and venlafaxine. The strain Ld-08 showed organic acids, ACC deaminase, phosphate solubilization, IAA, and siderophore. The sterilized bulbs of a *Lilium* variety, inoculated with Ld-08, were further studied for plant growth-promoting traits. The inoculated plants showed improved growth than the control plants. Importantly, some growth parameters such as plant height, leaf length, bulb weight, and root length were significantly (*P* ≤0.05) increased in the inoculated plants than in the control un-inoculated plants. Further investigations are required to explore the potential of this strain to be used as a plant growth-promoting and biocontrol agent in sustainable agriculture.

## Introduction

The plant-associated endophytic bacteria reside in various tissues where they contribute to plant growth promotion and fight against disease-causing agents [1]. The positive role of endophytic bacteria in the host is mainly governed by the endophytic ability to produce a diverse nature of enzymes, bioactive compounds, and secondary metabolites [2]. The metabolic activities of the endophytes confer the ability to survive in the complex chemical environment of the host and provide a selective advantage in terms of growth improvement and disease resistance [3,4]. Endophytes directly promote plant growth and development by producing of growth regulators such as indole acetic acid (IAA), phosphate solubilization, N-fixation, and 1-aminocyclopropane-1-carboxylic acid (ACC) deaminase. The indirect effects on plant health and its ability to cope with disease-causing pathogens may include the production of siderophore and the synthesis of bioactive compounds with antimicrobial properties [5,6].

Microbial technologies are expanding rapidly in agriculture mainly due to the isolation and characterization of new bacterial strains with enhanced growth-promoting and disease resistance potential [7]. *Pseudomonas aeruginosa* (PA) is a free-living soil bacterium that is also an opportunistic pathogen of both plants and animals. In plants, *P*. *aeruginosa* is the causal agent of a number of different diseases [8,9]. However, some species of the genus *Pseudomonas* have been extensively used in bioremediation, and as plant growth-promoting and biocontrol agents [10]. The *Pseudomonas* strains, isolated from diverse plant samples, have been proved very effective as plant growth-promoting and biocontrol agents and producers of antimicrobial compounds, antibiotics, enzymes, and volatile compounds [11,12]. Some strains of *Pseudomonas* such as *P*. *putida* WCS358, *P*. *fluorescens* WCS374, and *P*. *fluorescens* WCS417 were reported to enhance plant growth and protect plants against diseases [13]. The *Pseudomonas* strains, isolated from coastline soil samples, exhibited multiple plant growth-promoting attributes and showed biocontrol against phytopathogenic fungi [14]. Strains of *Pseudomonas* sp. isolated from the rhizospheric soil improved plant growth of tomato plants under greenhouse conditions [15]. Other studies reported similar plant growth-promoting and antifungal effects of the isolated strains of *Pseudomonas* sp. [16–18].

Sustainable agriculture production mainly relies on replacing the application of chemical fertilizers and pesticides with environment-friendly bio-inoculants based on endophytes and plant growth-promoting rhizobacteria (PGPR) [19]. There are several examples of endophytes that were successfully utilized in the commercial production of biofertilizers. Some endophytes, and their compounds, were used as natural insecticides and biopesticides [20]. Hence, the huge diversity of the plant-associated endophytic bacteria and their bioactive compounds necessitate further investigation and exploration of new endophytes with diverse plant growth-promoting and disease control properties. Medicinal plants, in particular, provide valuable resources of novel compounds that may be utilized as biocontrol agents against pathogenic diseases in crop plants [21]. In this connection, the bacterial endophytes associated with medicinal plants may be explored and utilized in plant growth promotion and disease resistance.

Members of the genus *Lilium* are widely distributed throughout China. *L*. *davidii* has considerable economic importance in China due to its food, health, and aesthetic uses [22]. Edible lily (*Lilium davidii* var. *unicolor*), an important member of the genus *Lilium*, has edible and medicinal uses [23–29]. In the present study, an endophytic bacteria isolated from the underground bulbs of *L*. *davidii* was investigated for antifungal, secondary metabolites, and plant growth-promoting effects.

## Materials and methods

### Isolation of endophytic bacteria

Freshbulbs of *L*. *davidii* were used for the isolation of endophytic bacteria. The bulbs were washed with tap water, and the individual bulblets were isolated. The peeled samples were washed and were sterilized with 70% (v ⁄ v) ethanol for 1 min, followed by 10% NaClO solution for 20 min. The bulbs samples were then washed with sterile distilled water and were cut aseptically into smaller pieces of approximately 1 cm x 1 cm sizes. The samples were placed on LB agar media and incubated at 30°C for 3 days. The bacterial colonies were selected, and sub-cultured in fresh LB broth and incubated at 30°C and pure cultures were obtained for further use.

### Morphological and microscopic characterization

The endophytic bacteria strains were characterized for morphological and microscopic properties. Morphological properties such as colony shape, color, and growth pattern were observed and recorded after 24 h of growth on LB. The gram staining reaction was conducted as previously described by Vincent and Humphrey [30]. The cell morphology of isolates was further characterized through S-3400N field-emission scanning electron microscope (Hitachi, Japan). The bacterial isolates in LB broth were cultured overnight at 30°C and shaking at 220 rpm. The culture was then centrifuged at 8000 rpm for 5 min and the pellet was rinsed with 0.2 M Phosphate buffer (pH 7.2–7.4), followed by fixing with 2.5% glutaraldehyde. The bacterial cell pellet was then washed with phosphate buffer and rinsed with sterile water. The pellet was further dehydrated with a concentration gradient of ethanol ranged from 30–100% for 15 min, at each step. After purification, the samples were mounted on microscopic coverslips and were observed for morphological features.

### Molecular characterization

The genomic DNA of the bacterial isolate was extracted using the “SolarBio” bacterial genomic DNA extraction kit. About 1500 bp product was amplified using the 16S rRNA gene-specific primers 27F (5´-AGAGTTTGATCCTGGCTGAG-3´) and 1378R (5´-CGGTGTGTACAAGGCCCGGGAACG-3´) [31]. The 25 μL PCR reaction contained 1 μL (0.5–10.0 ng) of template DNA, 0.5 μl (10 μM) of primers P027F and 1378R each, 12.5 μl of Premix Taq Version 2.0 (TaKaRa Bio Group), 10.5 μl of ddH_2_O. The PCR condition was set as initial denaturation at 94°C for 4 min, followed by 30 cycles of denaturation at 94°C for 30 s, 55°C for 1 min, and 72°C for 1 min, and a final extension at 72°C for 10 min. A 5 μL PCR product was run in the agarose gel (1%) at 100 volts for about 80 min. The amplified PCR product was gel purified using the QIAquick PCR Purification kit (Qiagen, Hilden, Germany). The amplified 16S rRNA gene product was sequenced (Beijing Biomed Gene Technology Co. Ltd). The sequence was then BLAST searched against the homologous 16S rRNA sequences in the NCBI GenBank database. The sequence was aligned with the homologous 16S rRNA gene sequences of other related species using "CLUSTALW". The phylogenetic tree was constructed based on the Maximum Likelihood (ML) algorithms in the MEGA 7 software [32].

### Antifungal activity

The antifungal activity of the isolated endophytic strains was tested *in vitro* against four pathogenic fungi, i.e., *F*. *oxysporum*, *B*. *cinerea*, *B*. *dothidea*, and *F*. *fujikuroi*. The antifungal tests were conducted using the dual culture method [33]. A 12 μL of two-days-old bacterial culture was spot inoculated at the four sides of the PDA plate. Then a plug of the pathogenic fungi was placed at the center of the PDA plate. Plates containing fungal plugs without bacterial inoculation were used as control. Plates were incubated at 30°C for 7 days. The fungal growth inhibition was calculated using the formula:

Percentage of growth inhibition = [(C − T)/C] × 100, where C is the radial growth of the test pathogen in the control plates (mm), and T is the radial growth of the test pathogen in the test plates (mm). The experiment was repeated twice with five plates per treatment.

### Ethyl acetate extraction of secondary metabolites

The bioactive secondary metabolites of the bacterial isolate were extracted using the solvent partition method. The isolated strain was cultured in LB broth at 30°C and 120 rpm for 6 days. The culture was then centrifuged at 12,000 rpm for 10 min at 4°C. The pellet was discarded, and the supernatant was filtered through a 0.2 μm syringe filter. The filtrate was mixed with an equal volume of ethyl acetate into the separating funnel. The mixture was shaken for 3 min, and the solvent phase containing the secondary metabolites was separated from the aqueous phase. The solvent was then evaporated to complete dryness to yield the crude extract. A sample of 20 mg crude extract was re-dissolved in 1 mL 70% methanol, and a 500 μL of this extract was filtered through a 0.2 μm filter. Samples were then analyzed through the ultrahigh performance liquid chromatography LTQ XL linear ion trap mass spectrometry/mass spectrometry (UHPLC-LTQ-XL-IT-MS/MS) system.

### UHPLC-LTQ-XL-IT-MS/MS analysis

UHPLC-LTQ-IT-MS/MS analysis was performed according to the method partially adapted from Lee et al. [34]. The Thermo Fisher Scientific LTQ XL linear ion trap mass spectrometry consisted of an electrospray interface (Thermo Fisher Scientific, San José, CA, USA) coupled with a DIONEXUltiMate 3000 RS Pump, RS Autosampler, RS Column Compartment (Dionex Corporation, Sunnyvale, CA, USA). The sample was separated on a Thermo Scientific Hypersil GOLD C18 column with a 1.9 μm particle size. The mobile phase consisted of A (0.1% (*v*/*v*) formic acid in water) and B (0.1% (*v*/*v*) formic acid in acetonitrile, and the gradient conditions were increased from 10% to 100% of solvent B. Scanning was set to start after 1 min to the source. Solvent gradient time was set over 19 min and re-equilibrated to the initial condition for 4 min by setting the divert valve to waste. The flow rate was set at 0.3 mL/min, and the injection volume was 10 μL. The temperature of the column during measurement was maintained at 35°C. Ion trap was performed in positive and full-scan ion modes within a range of 150–1000 *m*/*z*. The various operational parameters used were: source voltage, ±5 kV; capillary voltage, 39 V; capillary temperature, 275°C; auxiliary gas flow rate, 10−20 arbitrary units; sheath gas flow rate, 40−50 arbitrary units; and spray voltage 4.5 kV. Tandem MS (MS/MS) analysis was performed by scan-type turbo data-dependent scanning (DDS) under the same conditions for MS scanning for the six most intense ions using the Nth order double play mode. The sample analysis was performed for 3 biological replicates.The UHPLC-LTQ-IT-MS/MS data wereobtainedfromXcalibur software, Thermo Fisher Scientific.

The putative identification of secondary metabolites was conducted through molecular networking workflow from the GNPS website as previously described by Wang et al. [35]. ProteoWizard 3.0.19140 was used to convert the raw LC-MS files into mzXMLas described [36], and the mzXML file was then uploaded to GNPS. A molecular network was created using the default parameters and the spectra in the network were then searched against the GNPS spectral libraries. The library spectra were filtered in the same manner as the input data. All matches kept between network and library spectra were required to have a score above 0.7 and at least 6 matched peaks.

### Plant Growth-Promoting (PGP) assays

The plant growth-promoting properties of the isolated strain were determined through qualitative and quantitative tests. The bacterial strain was cultured in 1 mL LB broth at 30°C for 48 h and 200 rpm. The culture was centrifuged at 4000 rpm for 10 min. The cell pellet was washed thrice with 1 mL of 10 mM MgSO_4._ The pellet was then dissolved in 650 μL MgSO_4_ and was used for plant growth-promoting qualitative and quantitative tests.

### Organic acid production assay

The ability of the isolated strain to produce organic acids was tested according to the method described by Cunningham and Kuiack [37]. About 800 μL of Sucrose Tryptone medium (ST) was inoculated with 50 μL of the bacterial suspension in 10 mM MgSO_4._ The ST medium contained 20 g L^−1^ sucrose and 5 g L^−1^ tryptone and was supplemented with 10 mL of trace elements solution. The samples were incubated at 30°C and 200 rpm shaking for five days. Organic acids in the samples were recorded by adding 100 μL of 0.1% alizarine red S pH indicator, and the samples were further incubated for 15 min at room temperature. After incubation, the samples with yellow coloration were considered positive, while those with pink color indicated negative results for organic acid production.

### Phosphate solubilization assay

The phosphate-solubilizing potential of the isolated strain was evaluated as described by Mehta and Nautiyal [38]. The isolated endophytic bacterial strain was cultured on a solid NBRIP medium that contained 10 g L^-1^ glucose; 5 g L^-1^ Ca_3_(PO_4_)_2_; 5 g L^-1^ MgCl_2_; 0.1 g L^-1^(NH_4_)_2_SO_4_; 0.25 g L^-1^ MgSO_4_.7H_2_O; 0.2 g L^-1^ KCl; and 15 g L^-1^ agar. Ca_3_(PO_4_)_2_ was supplied as a sole source of inorganic phosphate. The halo and the bacterial colonies diameter on the NBRIP plates were measured every 24 h up to 4 days of incubation at 30°C. The solubilization index, defined as the ratio of the total diameter (colony + halo zone) to the colony diameter, was determined [39]. The P content was quantitatively estimated in the supernatant according to the vanado-molybdate colorimetric method [40].

### ACC deaminase production

The isolated strain’s 1-aminocyclopropane-1-carboxylate (ACC) deaminase activity was detected qualitatively according to the method [41]. A bacterial suspension of 250 μL in MgSO_4_ was added to 1.2 mL of salts minimal medium (SMN) containing 5 mM ACC as a sole source of N. After incubation at 30°C for 3 days, the cultures were centrifuged at 5000 rpm for 15 min, and the cell pellet was re-suspended in 100 μL of 0.1 M Tris–HCl buffer (pH = 8.5). The samples were then supplemented with 10 μL of 0.5 M ACC and 100 μL of 0.1 M Tris–HCl buffer (pH = 8.5) and then incubated at 30°C and 150 rpm for 30 min. After incubation, 150 μL of 0.2% 2, 4-dinitrophenylhydrazine reagent (in 2 N HCl) and 690 μL of 0.56 N HCl were added to the samples. The samples were again incubated at 30°C for 30 min, followed by the addition of 1 mL2 N NaOH. Samples without the addition of ACC were used as the negative control. Change of color from yellow to brown was regarded as positive for ACC deaminase production.

### IAA production assay

The production of indole acetic acid (IAA) in the isolated strain was determined according to the method of Gordon and Weber [42]. A 150 μL bacterial suspension prepared in 10 mM MgSO_4_ was inoculated in 3 mL of 1/10 diluted 869-rich medium for 5 days. Different flasks containing the 869-rich medium were inoculated with a loopful of the culture, and each flask was supplemented with various tryptophan concentrations, i.e., 0%, 0.2%, 0.4%, 0.6%, and 0.8%. The samples were incubated at a rotary shaker (150–180 rpm, 30°C). The production of IAA was measured after every 24 h interval. The bacterial cultures were harvested by centrifugation at 4000 rpm for 20 min, and about 1 mL of the supernatant was mixed with 2 mL of the Salkowski’s reagent (50 mL, 35% HClO_4_, 1 mL 0.5M FeCl_3_). A change of color from yellow to pink was considered positive. Optical density (OD) was measured at 530 nm, and the IAA quantities were determinedby comparing with the standard curve prepared with IAA concentrations. The experiment was repeated twice, each with three replicates.

### Nitrogen fixation

The ability of the isolated strain to fix atmospheric nitrogen was screened in a nitrogen-deficient malate medium (NFM). The *Escherichia coli* strain DH5α was used as a negative control. Plates containing the NFM medium were inoculated with colonies of the isolated strain and *E*. *coli*, DH5α. The NFM medium contained 0.01 g L^-1^ FeCl_3,_ 0.02 g L^-1^ CaCl_2_, 0.4 g L^-1^ KH_2_PO_4_, 0.1 g L^-1^ NaCl, 0.2 g L^-1^ MgSO_4_.7H_2_O, 0.002 g L^-1^ Na_2_MoO_4_.2H_2_O, 0.5 g L^-1^ K_2_HPO_4,_ 5 g L^-1^ sodium malate, 15 g L^-1^ agar, pH 7.2–7.4, and supplemented with yeast extract (50 mg/L) [43]. Growth of both the strain and *E*. *coli* was observed on NFM medium supplemented with NH_4_Cl. Plates were incubated at 28°C for 7 days. The experiment was repeated twice, each with three replicates.

### Siderophore detection assay

The production of siderophore was determined by forming an orange halo around bacterial colonies on Chrome Azurol S (CAS) agar plates incubated at 30°C for 48 h [44]. The CAS agar plates containing the bacterial colonies were incubated at 28°C for two weeks and the appearance of a yellow/orange halo around the bacterial colonies was observed. The isolated bacterium was cultured in liquid 284 medium with chrome azurol S (CAS) shuttle solution for quantatitive estimation. The bacterial suspension (50 μL) in 10 mM MgSO_4_ was added to 284 medium (800 μL), prepared with six different iron concentrations, i.e. 0 μM, 1 μM, 3 μM, 5μM, 7 μM, and 10 μM Fe(III) citrate. The samples were incubated at a rotary shaker (150 rpm, 30°C) for five days, followed by addition of 100 μL of the blue Chromium Azurol S (CAS) reagent to each sample and, incubation at room temperature for 4 h. The change of color from blue to orange/yellow was considered positive. The production of siderophore was further quantified by measuring the absorbance at 630 nm, and the activity was recorded in “percentage siderophore units” (psu) calculated as % of siderophore units = [(Ar–As) × Ar^-1^)× 100], where, "Ar" is the absorbance of reference (CAS reagent) at 630 nm; and "As" is the absorbance of the sample at 630 nm. Siderophore production was further confirmed through a qualitative test using CAS agar assay. All assays were carried out in triplicates.

### Pot experiment for assessment of plant growth-promotion

The ability of the isolated strain to promote plant growth was assayed using the Asiatic hybrid ’Tresor’ as the test variety under greenhouse conditions (25 ± 2°C and 70% RH). Bulbs with the same sizes and normal healthy appearance were selected for the pot experiment. The bulb samples were washed with tap water and were sterilized with 70% (v ⁄v) ethanol for 1 min, followed by treatment with 10% NaClO solution for 20 min. The isolated bacterial strain was cultured in 5 mL LB overnight and was re-cultured in 50 mL LB broth on a shaker (220 rpm, 30°C) for 24 h. After incubation, the culture was grown in 400 mL LB to the mid-log phase at 30°C for 24 h. The culture’s optical density (OD) was determined through a spectrophotometer and recorded as1.0 at OD600. For bulb inoculation, the bacterial culture was diluted 10 times with sterilized water. Bulbs of the ‘Tresor’ variety were inoculated in the diluted culture for about 40 min. The un-inoculated control bulbs were soaked for 40 min in simple LB broth, diluted 10 times. Soil pots having the size of 15 cm (bottom diameter), 21 cm (upper diameter), and 22 cm (height) were prepared with about 5 kg of soil mix of peat moss, perlite, and vermiculite (2:1:1) in the greenhouse. The inoculated and the un-inoculated bulbs of the ‘Tresor’ variety were sown in the soil pots having 3 bulbs in each pot. The pots were arranged in randomized block design. The experiment had one trial with two treatments, un-inoculated control, and bulbs inoculated with *P*. *aeruginosa* strain Ld-08. Each treatment had five soil pots (replicates), and each soil pot had three plantlets. Plants were watered with an equal amount of normal tap water every alternative day and were harvested after about four months (114 days) of vegetative and reproductive growth. At the time of harvest, various growth parameters such as shoot length, leaf length, leaf width, number of flowering stocks, stem diameter, root length, number of bulbs, and weight of bulbs were measured.

### Colonization assay

For colonization assay, the bulbs were surface sterilized with the same procedure as previously mentioned. The bulbs were inoculated with the overnight cultured bacterial strain Ld-08 (cell density of 10^7^ CFU/ml) for 40 min and were aseptically planted in small soil pots having two kg sterilized soil mix of peat moss, perlite, and vermiculite at a ratio of 2:1:1 in the greenhouse under same conditions previously described. Control bulbs were inoculated with sterilized phosphate buffer. After fifty days of sowing, the plants were harvested, and the roots were surface sterilized with 70% ethanol and 1% NaClO solution. For re-isolation of the bacterial endophyte, the macerated root samples were serially diluted in PBS, and 100 μL aliquots were spread on nutrient agar plates and incubated at 28°C for 3–4 days. After this period, the population size of the endophytic bacteria was measuredas CFU g^-1^ fresh root weight. The experiment was conducted in triplicate.

### Statistical analysis

The data were analyzed through analysis of variance (ANOVA). Means were compared with the student’s t-test at a probability of α = 0.05. The greenhouse data was subjected to ANOVA followed by student’s t-test with Bonferron’s correction. When the Bonerroni’s correction was applied, the p-values were transformed accordingly. A P-value of ≤ 0.05 was considered significant.

## Results

### Isolation and characterization of *Pseudomonas aeruginosa* Ld-08

**Fig 1:**
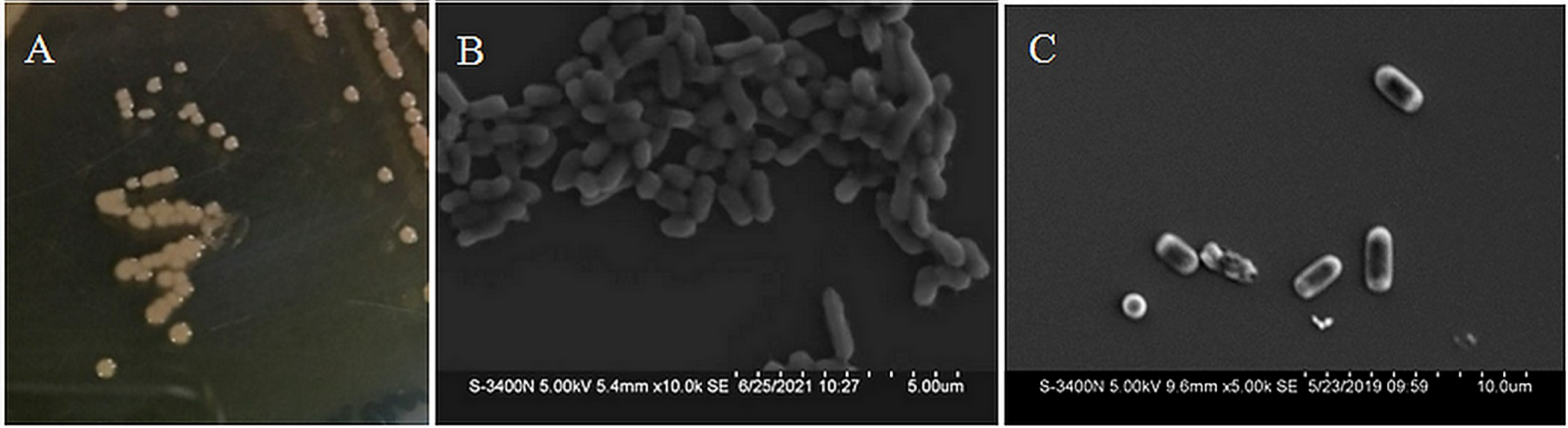
Colonies morphology and scanning electron microscope (SEM) analysis of the endophytic *P*. *aeruginosa* strain Ld-08, isolated from *L*. *davidii*. (A) Colony morphology of Ld-08 on LB agar plates. (B,C) SEM image showing rods-shaped structures.

**Fig 2:**
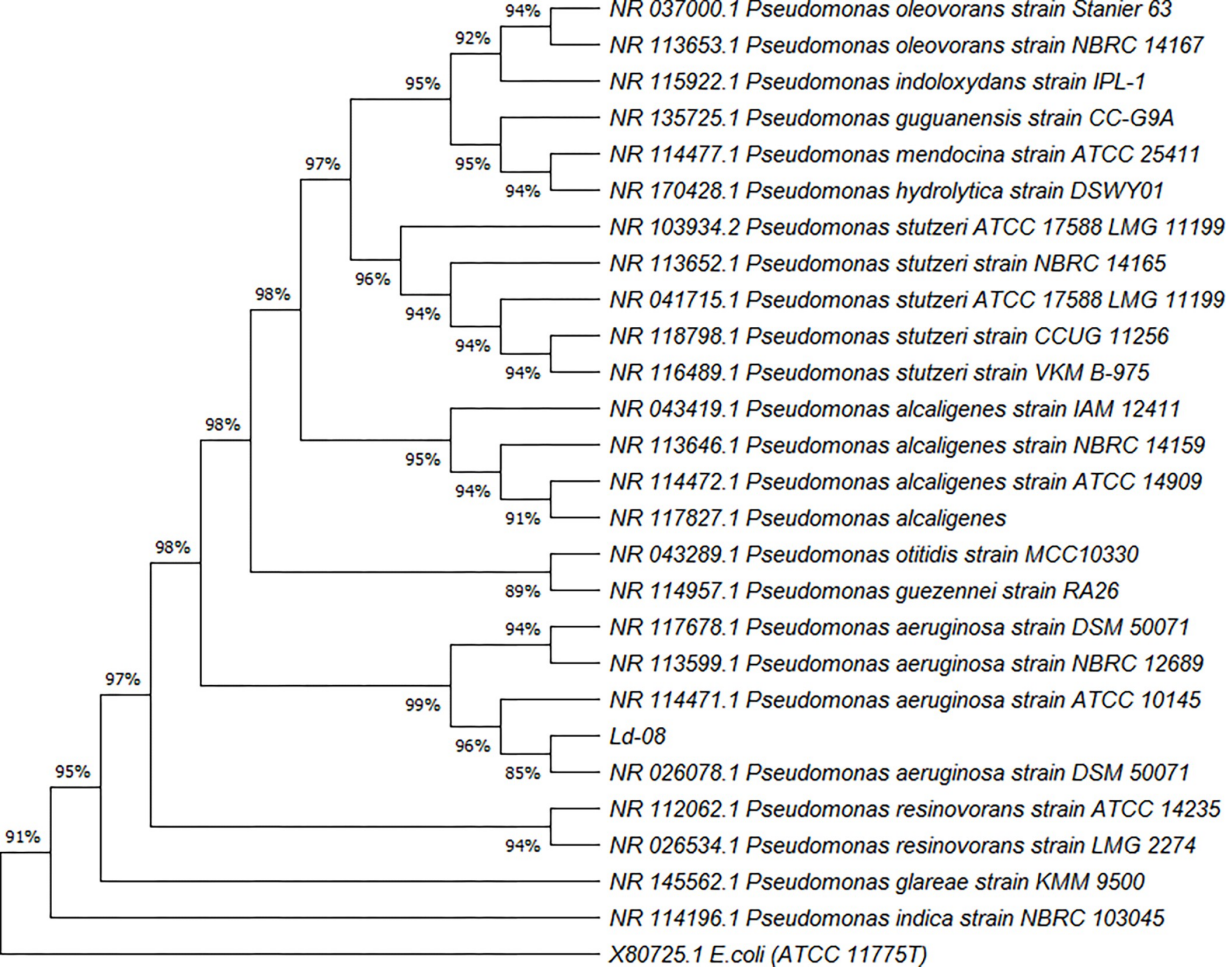
Maximum-likelihood phylogenetic tree based on the 16S rRNA gene sequences of strain Ld-08 and related strains in the genus *Pseudomonas*. Bootstrap values are shown as percentages of 1000 replicates. The *E*. *coli* strain ATCC 11775T was used as an out-group. Evolutionary analysis were conducted in MEGA X [45].

The endophytic isolate Ld-08 produced off-white round colonies on LB media plates (Fig 1A). The strain was identified as gram-negative and spore-forming bacterium. Morphological characterization through Scanning Electron Microscopic (SEM) analysis revealed rod-shaped structures (Fig 1B and 1C). Molecular and BLAST analysis of the 1400 bp long 16S rRNA sequence identified the isolated strain belonging to *Pseudomonas* genus. Based on the Maximum-likelihood phylogenetic tree, constructed with 16S rRNA gene similarity (%), the endophytic strain Ld-08 showed highest similarity with *P*. *aeruginosa* DSM 50071 (Fig 2). The 16S rRNA gene sequence of the isolated strain Ld-08 shared high similarities with other *Pseudomonas* species such as *Pseudomonas otitidis* MCC10330, *Pseudomonas* guezennei RA26, and *Pseudomonas resinovorans* LMG2274. The 16S rRNA gene sequence of Ld-08 was submitted to GenBank under accession no. MT472133.

### Antifungal assay

**Fig 3:**
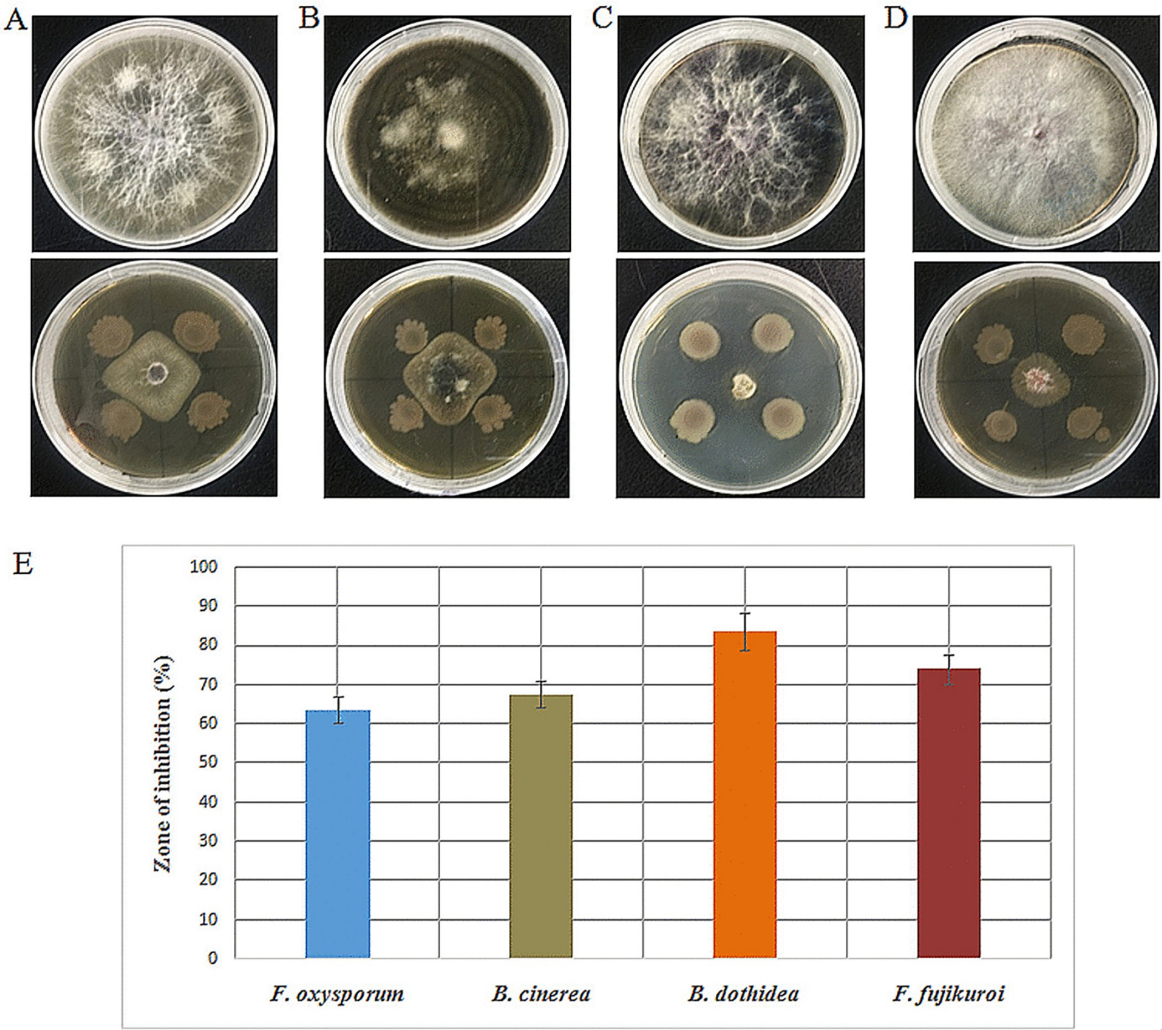
Antifungal activities of the isolated endophytic bacterial strain Ld-08 against fungal pathogens. A 5 mm fungus plug was inoculated into the center of the PDA medium surrounded by four spots of bacterial inoculum. (A), (B), (C), and (D) are control (upper) and test plates (lower) containing dual cultures of Ld-08 and the respective fungal pathogens, *F*. *oxysporum*, *B*. *cinerea*, *B*. *dothidea*, *and F*. *fujikuroi*, respectively. (E) antifungal activities were measured as the size of the zones of inhibition of the pathogenic fungi. Zones of inhibitions were expressed as percentages.

The endophytic Ld-08 strain showed high antifungal activity against the tested fungal pathogens. Four different fungal pathogenic strains, i.e., *F*. *oxysporum*, *B*. *cinerea*, *B*. *dothidea*, and *F*. *fujikuroi* were used in the present study. Antagonistic effects of the isolated strain Ld-08 were determined against the test fungal pathogens. The zones of inhibition of the test fungal pathogens were measured based on the dual culture plate assay. The *P*. *aeruginosa* Ld-08 showed considerable mycelial growth inhibition as revealed by the zones of inhibition that might be due to the release of diffusible compound (s) and secondary metabolites against the tested fungal pathogens (Fig 3A–3D). The highest percentage of growth inhibition i.e.83.82±4.76%, was observed against *B*. *dothidea*, followed by 74.12±3.87%, 67.56±3.35%, and 63.67±3.39% against *F*. *fujikuroi*, *B*. *cinerea*, and *F*. *oxysporum*, respectively (Fig 3E).

### Analysis of secondary metabolites

The endophytic strain *P*. *aeruginosa* Ld-08 was tested for the presence of bioactive compounds. Theethyl acetate fraction was investigated for metabolite profiling through UHPLC-LTQ-IT-MS/MS analysis. The secondary metabolites were putatively identified using the GNPS molecular networking workflow. The Thermo raw files were converted into mzXML using ProteoWizard 3.0.19140, and the mzXML files were uploaded to the GNPS. The spectra in the network were then searched against GNPS’ spectral libraries revealed that the tandem mass (MS/MS) spectrum of some compounds in the ethyl acetate fraction of *P*. *aeruginosa* Ld-08 was closely matched to the GNPS reference library (cosinescore above 0.7 and at least 6 matched peaks). The matched compounds were separated from those of the media control, and then a total of 30 compounds were putatively identified. An overview of the identified compounds are presented (Table 1). The intensities of all the detected peaks in the ethyl acetate fraction of Ld-08 were observed in the total ion chromatogram (S1 Fig). Some of the important bioactive secondary compounds and metabolites identified in the ethyl acetate fraction of Ld-08 included quinolones [2-(2-hepten-1-yl)-3-methyl-4-Quinolinol, 2-heptyl-3-hydroxy 4-quinolone, 1-methyl-2-nonylquinolin-4-one]; 3,9-Dimethoxypterocarpan; cascaroside B; dehydroabietylamine; epiandrosterone; nocodazole; oxolinic acid; pyochelin; rhodotulic acid; 9,12-octadecadienoic acid; di-peptides; tri-peptides; pinolenic acid methyl ester; ursodiol; and venlafaxine.

**Table 1:** Putative identification of secondary metabolites and compounds in the ethyl acetate fraction of *P*. *aeruginosa* Ld-08.

Compound name	Compound class	*m/z* measured	Library *m/z*	Molecular formula	Adduct	GNPS score	GNPS Library ID	CAS no.
2-(2-hepten-1-yl)-3-methyl-4-Quinolinol	Quinolones and derivatives	257.7	256.1	C_14_H_15_NO	M+H	0.759408	CCMSLIB00004679197	1052274-26-6
2-heptyl-3-hydroxy 4-quinolone	Quinolones and derivatives	260.6	260.0	C_16_H_21_NO_2_	M+H	0.942638	CCMSLIB00000006838	108985-27-9
4-hydroxy-2-heptylquinoline N-oxide	4-hydroxy-2-alkylquinolines	260.3	260.0	C_16_H_21_NO_2_	M+H	0.790237	CCMSLIB00000006840	N/A
1-Methyl-2-[(6Z)-6-undecen-1-yl]-4(1H)-quinolinone	Quinolones and derivatives	312.9	312.2	C_21_H_29_NO	M+H	0.811881	CCMSLIB00000855673	120693-49-4
1-methyl-2-nonylquinolin-4-one	Quinolones and derivatives	284.7	286.2		M+H	0.78857	CCMSLIB00000845108	n/a
4-[(6-methoxyquinolin-8-yl)amino]-4-oxobutanoic acid	Quinolines and derivatives	274.0	275.1	C_14_H_14_N_2_O_4_	[M+H]+	0.727704	CCMSLIB00000578876	N/A
Oxolinic Acid	Quinoline carboxylic acids	260.3	262.0	C_13_H_11_NO_5_	[M+H]+	0.791188	CCMSLIB00000562778	N/A
(2R,3R,4S,5S,6R)-2-[(3R)-1,7-bis(3,4-dihydroxyphenyl)heptan-3-yl]oxy-6-(hydroxymethyl)oxane-3,4,5-triol	Linear diarylheptanoids	494.3	493.2	C_25_H_34_O_10_	[M-H]-	0.726848	CCMSLIB00004697442	N/A
3,9-Dimethoxypterocarpan	Furanoisoflavonoids	284.5	283.1	C_17_H_16_O_4_	M-H	0.743852	CCMSLIB00004686015	N/A
Cascaroside B	Carbohydrates and carbohydrate conjugates	604.6	603.0	C_27_H_32_O_14_	M+Na	0.856945	CCMSLIB00000574567	
LL-2,6-diaminoheptanedioate	Amino acids, peptides, and analogues	188.7	189.0	C_7_H_14_N_2_O_4_	M-H	0.72268	CCMSLIB00000578156	583-93-7
Dehydroabietylamine	Diterpenoids	285.0	286.2	C_20_H_31_N	[M+H]+	0.772666	CCMSLIB00000204891	1446-61-3
Estriol	Estrane steroids	272.6	271.2	C_18_H_24_O_3_	[M-H_2_O+H]+	0.861238	CCMSLIB00000213308	50-27-1
Epiandrosterone	Androstane steroids	274.3	273.3	C_19_H_30_O_2_	[M-H_2_O+H]+	0.702668	CCMSLIB00000213405	481-29-8
Nocodazole	Benzimidazoles	303.6	302.0	C_14_H_11_N_3_O_3_S	M+H	0.727357	CCMSLIB00000085358	N/A
Phenazine-1-carboxylic acid	Benzodiazines	224.6	225.0	C_13_H_8_N_2_O_2_	M+H	0.943749	CCMSLIB00000072150	N/A
pyochelin	Amino acids, peptides, and analogues	325.5	325.0	C_14_H_16_N_2_O_3_S_2_	M+H	0.875475	CCMSLIB00000006841	69772-54-9
Rhodotulic acid	Amino acids, peptides, and analogues	346.6	345.2	C_14_H_24_N_4_O_6_	M+H	0.750809	CCMSLIB00001059092	
12R-Hydroxy-5Z,8Z,10E,14Z-eicosatetraenoic acid	Eicosanoids	302.3	303.2	C_20_H_32_O_3_	M+H-H_2_O	0.75422	CCMSLIB00003140157	82337460
9(11),16,(5.alpha.)-Pregnadien-3.beta.-ol-20-one	Oxosteroids	627.4	629.4	C_21_H_30_O_2_	2M+H	0.788716	CCMSLIB00003137400	16300804
9,12-Octadecadiynoic acid	Fatty acids and conjugates	260.1	259.2	C_18_H_28_O_2_	M+H-H_2_O	0.797234	CCMSLIB00003135952	2012148
Arg-Asn	Amino acids, peptides, and analogues	290.9	289.1	10H_20_N_6_O_4_	M+H	0.784109	CCMSLIB00003136310	N/A
Leu-Pro	Amino acids, peptides, and analogues	210.9	211.1	C_11_H_20_N_2_O_3_	M+H-H_2_O	0.948916	CCMSLIB00003139607	N/A
Phe-Pro	Amino acids, peptides, and analogues	263.1	263.1	C_14_H_18_N_2_O_3_	M+H	0.971566	CCMSLIB00003139670	N/A
Ile-Pro-Ile	Amino acids, peptides, and analogues	342.4	342.2	C_17_H_31_N_3_O_4_	M+H	0.903765	CCMSLIB00003139778	90614485
Phe-Pro-Lys	Amino acids, peptides, and analogues	196.3	196.1	C_20_H_30_N_4_O_4_	M+2H]	0.872113	CCMSLIB00003136290	N/A
Ethanol, 2-(2-butoxyethoxy)	Ethers	162.8	163.1	C_23_H_28_N_2_O_5_	M+H	0.92388	CCMSLIB00003137269	112345
Pinolenic acid methyl ester	Lineolic acids and derivatives	244.3	243.2	C_19_H_32_O_2_	243.2	0.808745	CCMSLIB00003135592	38406574
Ursodiol	Bile acids, alcohols and derivatives	376.6	375.2	C_24_H_40_O_4_	M+H	0.71938	CCMSLIB00000005521	N/A
Venlafaxine	Anisoles	277.2	278.2	C_17_H_27_NO_2_	M+H	0.732527	CCMSLIB00000004418	N/A

### ACC deaminase, organic acids production and phosphate solubilization assay

**Fig 4:**
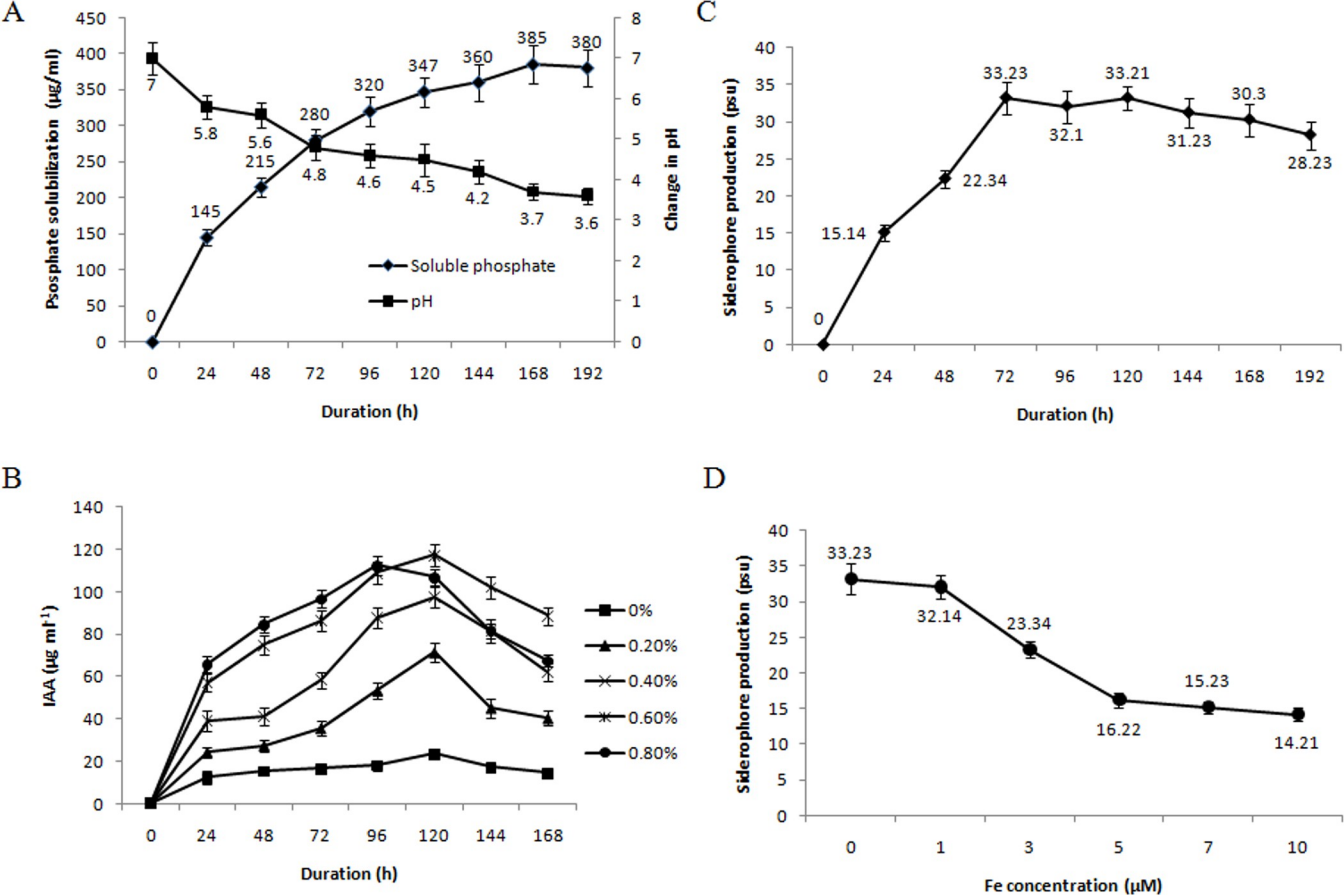
PGP effects of Ld-08. (A) Phosphate solubilization after different time intervals. Soluble free phosphate concentration is given against the primary y-axis, while a change of pH in the culture medium is given at the secondary y-axis. Standard deviations showed as bars. (B) Quantitative estimation of IAA produced by Ld-08 at different L-tryptophan concentrations. (C) Quantitative estimation of siderophore release against growth of bacteria. (D) Effect of iron source on the production of siderophore.

The strain Ld-08 was tested for production of ACC deaminase through a qualitative test. The endophytic strain Ld-08 exhibited production of ACC deaminase in moderate quantities, as revealed by the orange color strength (S2A Fig). The ability of the endophytic strain Ld-08 to produce organic acids was assayed through a qualitative test. The Ld-08 strain showed the potential of organic acids production in sucrose tryptone (ST) mediumas revealed by the change of color from pink to yellow upon addition ofalizarine red S pH indicator (S2B Fig). The organic acid detection was followed by the analysis of the phosphate solubilizing activity. The endophytic strain Ld-08 solubilized tri-calcium phosphate in the solid NBRIP medium, forming clear halos around the bacterial colonies (S3A Fig). The phosphate solubilization potential of the strain increased with increasing the culture time from 24 h to 168 h. During this time, the solubilized phosphate ranged from 145–385 μg mL^-1^. However, further incubation decreased phosphate solubilization potential. The pH of the medium decreased with an increase in the incubation time, and the amount of free phosphate was released, showing maximum phosphate solubilization at pH 3.7 after 168 h of incubation (385 μg mL^-1^) (Fig 4A). The correlation coefficient (r) between free phosphate concentrations against pH after various durations were found to be (−)0.9597.

### Indole Acetic Acid (IAA) detection

The strain Ld-08 produced IAA as evident from the change of color of the culture supernatant from yellow to pink (S2C Fig). The IAA was quantified in the culture medium of Ld-08 at various exogenously supplied tryptophan concentrations i.e. 0%, 0.2%, 4%, 6%, and 0.8%. The IAA production by Ld-08 increased in the culture medium with increasing the tryptophan concentrations and incubation time (Fig 4B). Maximum IAA production (117.4±5.3 μg mL^-1^) was observed at 0.6% tryptophan concentrationat 120 h of incubation. After this incubation period, the IAA production declined with further incubation. A similar trend was observed for all tryptophan concentrations except 0.8%, where maximum IAA (112.5±4.7 μg mL^-1^) was accumulated by Ld-08 after 96 h of incubation. These results indicated that the isolated *P*. *aeruginosa* Ld-08 has a high potential of IAA production with or without exogenous tryptophan application. However, the exogenous tryptophan application significantly increased the IAA production ability of Ld-08, and a positive correlation was observed between the various tryptophan concentrations and the accumulated IAA contents.

### Nitrogen fixation

The isolated strain Ld-08 was further tested for its ability to fix atmospheric nitrogen. The nitrogen-fixation potential of Ld-08 was tested by observing the growth on nitrogen-free minimal medium (NFM) (S3C Fig). *E*. *coli* strain DH5α was used as a negative control because this strain cannot grow on a nitrogen-free medium. First, the two strains were co-cultured on NFM medium, and it was observed after five days of incubation that Ld-08 was able to grow on the medium, while the *E*. *coli* strain failed to grow. Both strains were then cultured on an NFM supplemented with 5 mM NH_4_Cl, a preferred source of nitrogen. Our results showed that both strains grew on nitrogen supplemented medium and produced visible and clear colonies. These results revealed the nitrogen-fixation potential of Ld-08.

### Siderophore production

Siderophore production in Ld-08 was assayed through qualitative and quantitative assays. The strain Ld-08, cultured in liquid 284 medium with chrome azurol S (CAS) shuttle solution, confirmed production of siderophore through a change of color from blue to orange-yellow (S2D Fig). The siderophore production was further confirmed through chrome azurol S (CAS) on agar plates. The endophytic strain Ld-08 produced a clear orange/yellow halo zone around the bacterial colonies, confirming siderophore production (S3B Fig). The change of color of the CAS medium around the bacterial colonies indicated the ability of Ld-08 to produce siderophore. The size of the yellow/orange halo zone averaged 8.9±0.8 mm in diameter.

Siderophore production in Ld-08 was quantified in the medium against the growth of the bacterium and was confirmed by the CAS test, where the decolorization of CAS reagent from blue to orange was observed. Siderophore production in Ld-08 started after 2–3 hours of incubation in liquid 284 medium, and maximum siderophores were released after 72 h of incubation (33.23±2.1 psu) (Fig 4C). It seemed the siderophore production was maximum at the late log phase, after which a gradual decline was observed as the incubation time increased to 192 h. Further, the effect of different iron concentrations in the culture medium was investigated on the siderophore production ability of Ld-08. The strain was incubated in 284 medium supplemented with different iron (Fe(III) citrate) concentrations, i.e., 0 μM, 1 μM, 3 μM, 5 μM, 7 μM, and 10 μM. Iron availability in the medium significantly affected siderophore production. The endophytic strain Ld-08 showed high siderophore production when cultured in liquid 284 medium at 0 μM iron,i.e., 33.23±2.1 psu (Fig 4D). However, a gradual decline in the siderophore production was observed when the iron concentration increased from 1 μM to 10 μM Fe(III) citrate in the culture medium. The siderophore production significantly dropped to 16.22±1.1 psu when the iron concentration in the medium was raised from 1 μM to 5 μM. Beyond 5 μM iron concentration, a slight reduction in siderophore production was observed, and a total of 14.21±0.9 psu siderophore was accumulated by Ld-08 at 10 μM iron concentration in the medium.

### Pot experiment for endophytic colonization and assessment of plant growth-promotion

As the isolated strain, Ld-08 exhibited multiple plant growth-promoting traits and was resistant to the growth of different fungal pathogens; a pot test in the greenhouse was designed to determine the impact of Ld-08 inoculation on the overall growth of the Asiatic hybrid ’Tresor’. Before cultivation in soil pots in the greenhouse, the bulbs were inoculated with the isolated strain *P*. *aeruginosa* Ld-08. Several plant growth parameters, i.e., plant height, number of flowering shoots, leaf width, leaf length, stem diameter, bulb weight, and root lengths were measured between the inoculated and un-inoculated control plants upon completion of vegetative and reproductive growth. Further, the isolated strain Ld-08 to colonize the roots of the inoculated plants was tested. Our results showed that the endophytic bacterial strain Ld-08 successfully colonized the plant roots of ‘Tresor’ variety with a higher density of 4.80 log_10_ CFUg^-1^ fresh weight.

**Fig 5:**
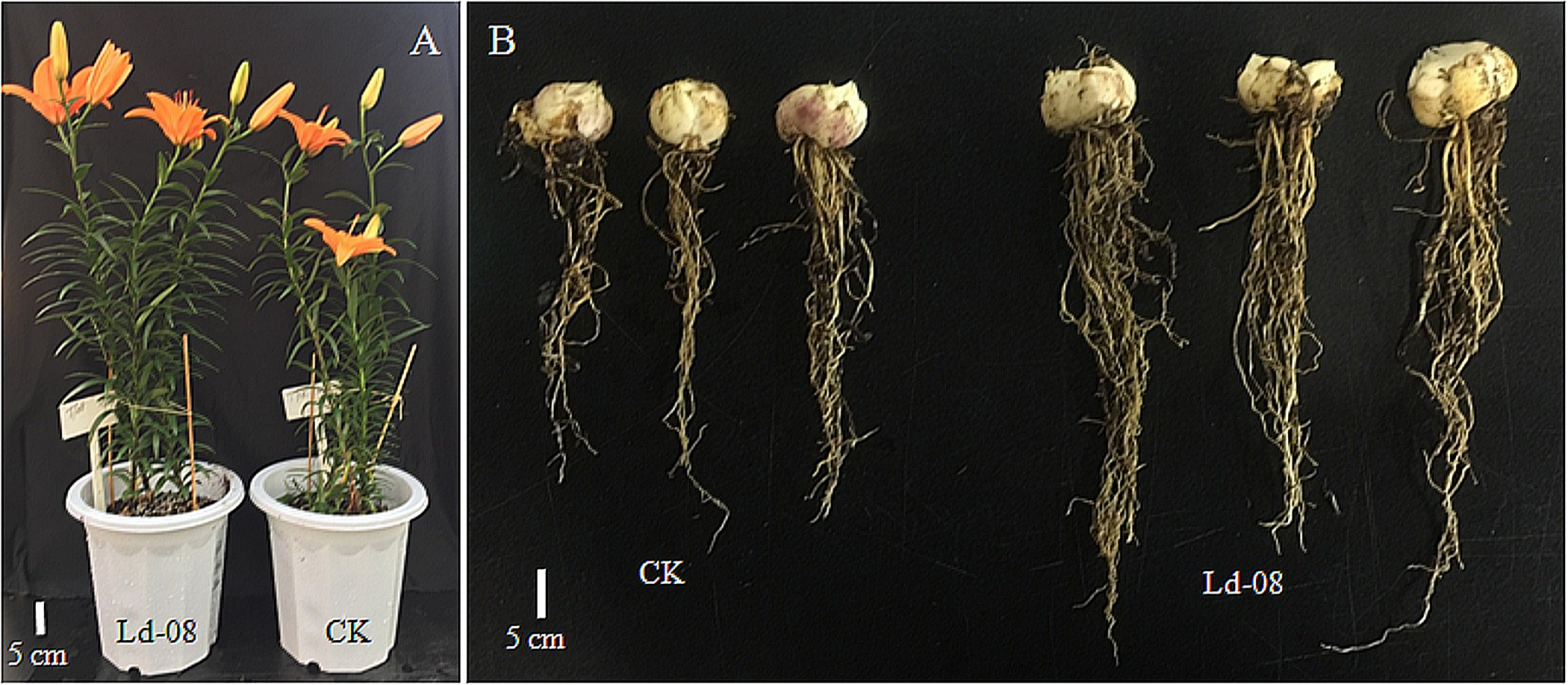
Plant growth promotion in *Lilium* variety “Tresor” upon inoculation of bulbs with Ld-08. (A) Relative plant height of Ld-08-inoculated and control plants (CK). (B) Relative root lengths of inoculated and control plants.

Inoculation of the ‘Tresor’ bulbs with Ld-08 positively impacted the tested parameters, and some growth attributes were significantly improved in the inoculated plants. The inoculation plants showed an overall improved vegetative and reproductive growth compared to un-inoculated control plants (Table 2). Inoculation of bulbs with Ld-08 resulted in a considerable increase in plant height (Fig 5A). The ’Tresor’ variety showed significantly higher (p≤0.05) plant height, i.e., 50.3±3.6 cm compared to 44.8±3.2 cm in the control plants (Table 2). Likewise, other growth parameters such as leaf length and root length were significantly affected between inoculated and non-inoculated control plants. The inoculated plants showed significantly greater (p≤0.05) leaf length, i.e., 108.3±6.8 mm, compared to 89.2±8.7 mm in control plants. The inoculation of bulbs with Ld-08 showed improvement in other traits such as leaf width, stem diameter, and flowering shoots; however, the differences were not significantly different.

**Table 2:** Plant growth-promoting effects of *Pseudomonas aeruginosa* Ld-08 on ‘Tresor‘ variety.

Treatments	Plant height (cm)	Leaf length (mm)	Leaf width (mm)	# of flowering shoots	Stem diameter (mm)	Root length (cm)	Bulb weight (g)
CK	44.8±3.2^a^	89.2±8.7^a^	9.7±1.4^a^	2.7±0.4^a^	8.9±0.7^a^	16.9±2.4^a^	17.0±1.8^a^
Ld-08	50.3±3.6^b^	108.3±6.8^b^	10.9±1.0^a^	2.9±0.4^a^	9.3±1.0^a^	26.4±2.5^b^	23.8±2.4^b^

Means are averages ± standard deviations (SD). Values in a column with different letters are significantly different (P-value ≤ 0.05) according to ANOVA and student´s t-test with Bonferroni´s correction for multiple comparisons.

Moreover, a significant impact of inoculation was observed on the growth of roots and the development of bulbs. The inoculated plants developed significantly (p≤0.05) thicker and longer roots (26.4±2.5 cm) than that of the control plants (16.9±3.5 cm) (Table 2 and Fig 5B). Significant differences were also found in the weight of bulbs between the inoculated and non-inoculated plants. The inoculated plants showed an average bulb weight of 23.8±2.4 g as compared to 17.0±1.8 g of control plants. The pot experiment results demonstrated the multiple plant growth-promoting potential of the isolated *P*. *aeruginosa* Ld-08.

## Discussion

Endophytic bacteria are considered an important bioresource with immense potential to confer the host plants the ability to grow better under normal and biotic or abiotic stress conditions. Plant growth-promoting rhizobacteria (PGPR) and endophytic bacteria associated with medicinal plants have been focused on due to antipathogenic and health-promoting secondary metabolites in these microbes [21,46]. An endophytic bacteria, *P*. *aeruginosa* Ld-08, was isolated from the bulb part of *L*. *davidii*. The strain was identified and characterized through electron microscopy and molecular phylogenetic analysis.

The isolated strain Ld-08 exhibited antagonistic effects against several fungal pathogens like *F*. *oxysporum*, *B*. *cinerea*, *B*. *dothidea*, and *F*. *fujikuroi* and inhibited their mycelia growth in a dual culture plate method. These fungal pathogens have been reported to cause serious diseases in crop plants [47,48]. The isolated endophytic bacteria *P*. *aeruginosa* Ld-08 was found to have the potential to inhibit the mycelia growth of the tested fungal pathogens. Earlier studies have reported the biocontrol potential of *Pseudomonads* against phytopathogens. Shanmugaiah et al. [49] reported the antifungal activity of *P*. *aeruginosa* MML2212 against *Rhizoctonia solani*. The metabolites produced by *P*. *aeruginosa* inhibited the mycelia growth of *R*. *solani*. Moreover, the *Pseudomonas* sp. was used to control the broccoli root rot disease caused by *R*. *solani* [50]. *P*. *aeruginosa* identified from the mine soil sample showed antagonistic activity against broad-spectrum phytopathogens [51]. *Pseudomonas* sp. isolated from a soil sample exhibited inhibition of mycelia growth of several grapevine fungal pathogens, including *B*. *cinerea* [17]. The broad-spectrum antifungal activity of the strain was attributed to the presence of three genes, *phlD*, *pltB*, and *prnC*, responsible for the synthesizing of antifungal compounds 2,4-diacetylphloroglucinol (2,4-DAPG), pyoluteorin, and pyrrolnitrin, respectively.

In the present study, the isolated endophytic strain Ld-08 was assayed for the secondary metabolites in the ethyl acetate fraction. Several bioactive compounds and secondary metabolites with potential antifungal properties were detected. The important bioactive compounds were quinolones [2-(2-hepten-1-yl)-3-methyl-4-Quinolinol, 2-heptyl-3-hydroxy 4-quinolone, 1-methyl-2-nonylquinolin-4-one]; 3,9-Dimethoxypterocarpan; cascaroside B; dehydroabietylamine; epiandrosterone; nocodazole; oxolinic acid; pyochelin; rhodotulic acid; 9,12-octadecadienoic acid; di-peptides; tri-peptides; pinolenic acid methyl ester; ursodiol; and venlafaxine. The presence of these bioactive secondary metabolites in the ethyl acetate fraction of Ld-08 might be responsible for the broad-spectrum antifungal activities of the strain. Most of the identified compounds in the ethyl acetate fraction of Ld-08 were previously reported as antimicrobial agents. The quinolone-type compounds produced in *P*. *aeruginosa* have long been considered antimicrobial properties [52]. These compounds were reported to act as signaling molecules in regulating the expression of multiple virulence genes in *P*. *aeruginosa*. The 2-heptyl-3-hydroxy 4-quinolone was reported to possess iron chelation and antimicrobial activities [53]. In *Pseudomonas*, the quinolone-type compounds were reported to function in quorum sensing, cytotoxicity, iron acquisition, and resistance against disease-causing phytopathogens [54]. Pterocarpans play animportant role as phytoalexins and constitute the second-largest group of natural isoflavonoids. The antifungal properties of pterocarpans in several studies are reviewed [55]. Cascaroside B, putatively identified in the present study, is a well-known flavonoid and was previously reported to possess antifungal properties [56]. The antifungal activities of dehydroabietylamine, epiandrosterone, and nocodazolewere previously reported [57–59]. Pyochelin, reported in the present study, was previously known as an iron chelator produced in endophytes. It may also play an important role in plant resistance to pathogens [60]. The reported compounds in the present study, i.e., rhodotulic acid; 9,12-octadecadiynoic acid; pinolenic acid methyl ester; and ursodiol, were previously reported with antifungal and antibacterial effects [61–64].

Endophytic bacteria promote the growth of associated plants through the expression of various plant growth-promoting traits such as the production of ACC deaminase, organic acids, indole acetic acid, siderophores, and nitrogen assimilation and phosphate solubilization [65]. Likewise, the isolated strain Ld-08 showed multiple plant growth-promoting traits. The Ld-08 strain showed production of organic acids, detected through a qualitative test. Previous studies demonstrated that the production of organic acids is one of the key mechanisms utilized by endophytes to promote the growth of associated plants and defense against disease-causing pathogens [66]. The production of organic acids is a sign that the endophytic bacteria can solubilize inorganic phosphate [67]. In the present study, the Ld-08 strain produced a halo zone on the NBRIP solid medium containing tri-calcium phosphate. The pH of the medium decreased with an increase in the amount of free phosphate released, showing maximum phosphate solubilization (385 μg/mL) at pH 3.7 after 168 h of incubation. Somewhat similar results were produced in earlier studies. The rhizobacterial strains *Pantoea cypripedii* and *Pseudomonas plecoglossicida* solubilized 253 μg mL^-1^ and 271 μg mL^-1^ of inorganic phosphate, respectively after 120 h of incubation [68]. Devi et al. [69] reported that the isolated *P*. *aeruginosa* strain AL2-14B had solubilized maximum phosphate of 383 μg mL^-1^ at pH 3.9 after 144 h of incubation.

Moreover, the Ld-08 strain exhibited production of ACC (deaminase), detected through a qualitative test. ACC deaminase has an antagonistic effect against ethylene production in plants. Ethylene is a signaling molecule that lowers plant growth during biotic and abiotic stresses [70]. Previous studies reported that inoculation of plants with ACC deaminase lowered the ethylene levels and thus reduced the growth inhibition during stress [71]. The ACC deaminase production was reported by *Bacillus* and *Pseudomonas* species with plant growth-promoting effects in previous studies [69,72].

The isolated strain Ld-08 released IAA in the medium, and its concentration was induced by L-tryptophan in the medium, suggesting the strain release IAA in tryptophan dependent mechanism, which was in agreement with previous reports [73]. Maximum IAA concentration was released on supplementation of 0.6% tryptophan after 120 h of incubation, which was in agreement with previous studies [74]. Fouzia et al. [75] reported an increase in IAA release by isolated *Pseudomonas* species on supplementation of L-tryptophan in the medium. Moreover, the isolated *P*. *aeruginosa* strain AL2-14B showed maximum IAA release after 96 h of incubation on supplementation of 1.0% L-tryptophan [69]. The endophytic Ld-08 strain grew on a nitrogen-free medium (NFM), and the growth was induced on NFM supplemented with NH_4_Cl. Previous studies had reported N-fixing endophytic *Pseudomonas* species from maize and sugarcane [76,77]. Devi et al. [69] reported the N-fixing potential of *P*. *aeruginosa* strain, isolated from *Achyranthes aspera* L.

The siderophore production ability of endophytic bacteria contributes to enhancing plant growth and yield potential [78]. The endophytic *P*. *aeruginosa* Ld-08 strain produced a significant amount of siderophore. About 33.23±2.5 unit of siderophore was recorded in succinate broth after 72 h of incubation at the onset of the late log phase. Further incubation time decreased the siderophore concentrations. Iron supplementation to the medium harmed the siderophore release. A steady decrease in siderophore release was observed when iron concentration was increased, which indicates that the Ld-08 strain release siderophore under the tight control of iron concentration. Our results agree with earlier studies, where an inverse relationship was observed between siderophore production by *P*. *aeruginosa* strains and different iron concentrations [74,79,80].

There are no previous reports where *P*. *aeruginosa* had been isolated from *Lilium* species. Here, we report an endophytic *P*. *aeruginosa* strain Ld-08, isolated from the bulb portion of *L*. *davidii*. Inoculation of *Lilium* variety, the Asiatic hybrid ‘Tresor’ with Ld-08 promoted vegetative and reproductive growth as confirmed by pot trial experiment. The inoculation experiment suggested that Ld-08 is a promising growth promoter of ‘Tresor’, as some growth parameters such as plant height, leaf length, bulb weight, and root length were significantly (*P* ≤ 0.05) increased. Some earlier studies reported *P*. *aeruginosa* strains, isolated from various plants, as plant growth-promoting [81,82]. In the present study, the potted soil, was not supplemented with any fertilizer or pesticide, which could promote the growth of plants. Therefore, the growth improvement of inoculated plants might be attributed to the endophytic colonization of *P*. aeruginosa Ld-08 with growth-promoting attributes including antifungal potential, IAA, and siderophore production, nitrogen fixation, and phosphate solubilization. The overall mechanism of growth improvement of inoculated plants has been attributed to the combined effects of various physiological properties of the endophytic bacteria, including the synthesis of antifungal compounds, phytohormones, siderophores, and ability of nitrogen fixation and phosphate solubilization [83], which is also valid for endophytic strain Ld-08.

## Conclusions

The endophytic *P*. *aeruginosa* Ld-08, isolated from the bulbs of the *L*. *davidii* plant, showed antifungal activities and produced a sufficient quantity of IAA, solubilized inorganic phosphate, and siderophore. The ethyl acetate extraction analysis detected several bioactive compounds and secondary metabolites with potential antifungal properties. Inoculation of *Lilium* variety ‘Tresor’ with Ld-08 performed better in some growth parameters as compared to those of non-inoculated control plants. This positive impact on the inoculated plants might be due to the plant growth-promoting nature of Ld-08. However, before utilizing Ld-08 as a biofertilizer and biocontrol agent, its antifungal nature and potential to confer disease resistance in the inoculated plants may require further studies.

## Supporting information

S1 Fig The TIC Chromatogram of the ethyl acetate fraction of endophytic strain *P. aeruginosa* Ld-08. (TIF)

S2 Fig Qualitative analysis of plant growth-promoting traits of Ld-08. (A) ACC deaminase activity. The upper well with brown coloration showed ACC deaminase detection while the lower well with yellow color was used as negative control. (B) Organic acids production as revealed by a color change to yellow in the upper well, while extreme lower well with pink color was used as negative control. (**C**) Detection of IAA showing a change of coloration from yellow to pink. (D) Siderophore production was confirmed by a change of color from blue to yellow/orange.
(TIF)

S3 Fig Analysis of phosphate solubilization, siderophore and nitrogen-fixation on plates. (A) Zone of clearance around colonies of Ld-08 confirming its role in phosphate solubilization. (B) Siderophore release and halo zone formation in CAS agar medium. (C) Nitrogen-fixation assay of Ld-08 strain on nitrogen-deficient malate medium (NFM) and was assessed for growth in reference to non-nitrogen fixing *E*. *coli* DH5α on NFM medium, and NFM supplemented with 5 mM NH_4_Cl.
(TIF)
