# Supplementary figures and images for: Pseudomonas aeruginosa Ld-08 isolated from Lilium davidii exhibits antifungal and growth-promoting properties
Source: PLoS One. 2022 Jun 17;17(6):e0269640. doi: 10.1371/journal.pone.0269640 (PMC9205524; doi:10.1371/journal.pone.0269640)

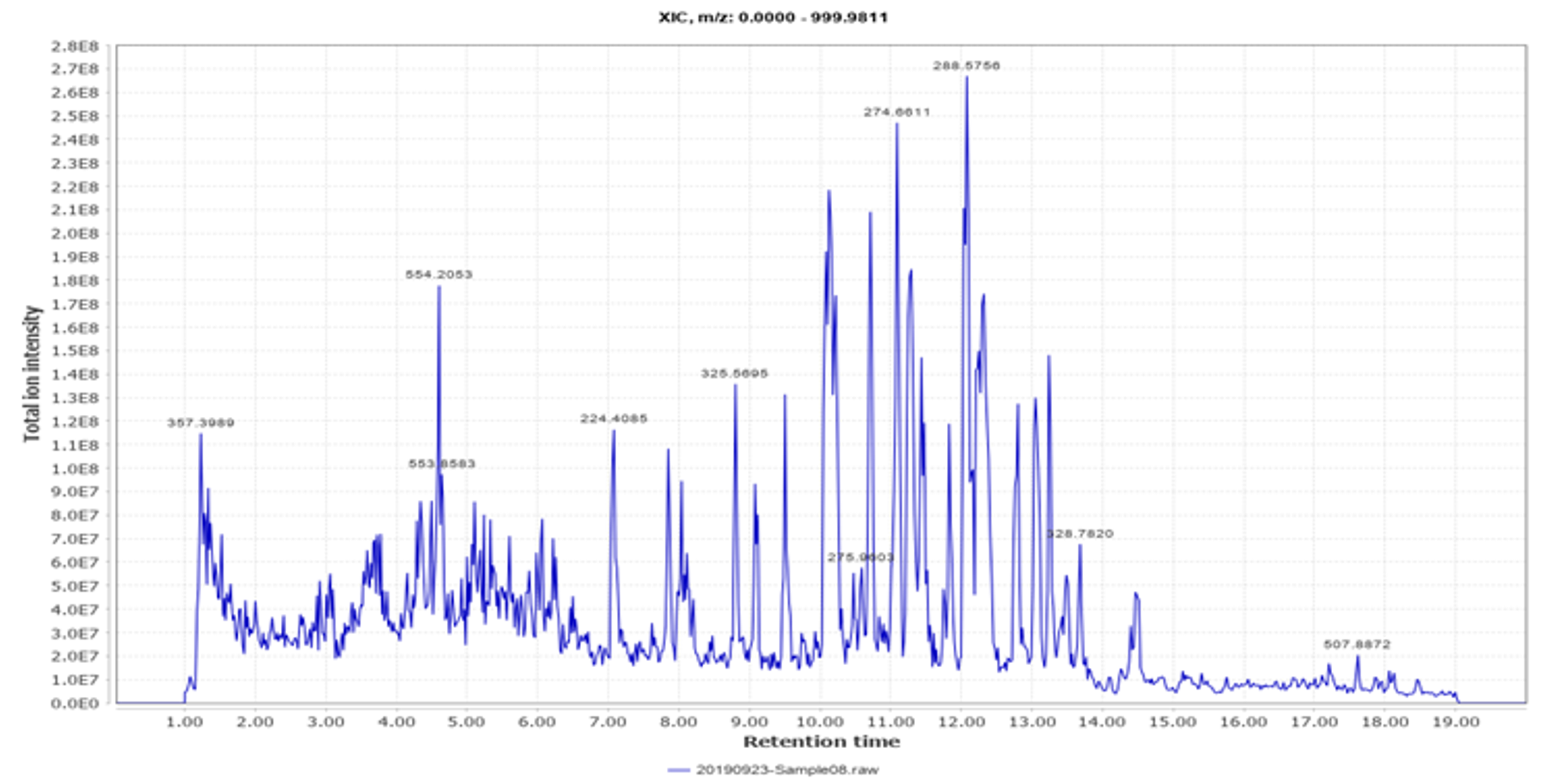

Supplement: S1 Fig — (TIF) [file pone.0269640.s001.tif]

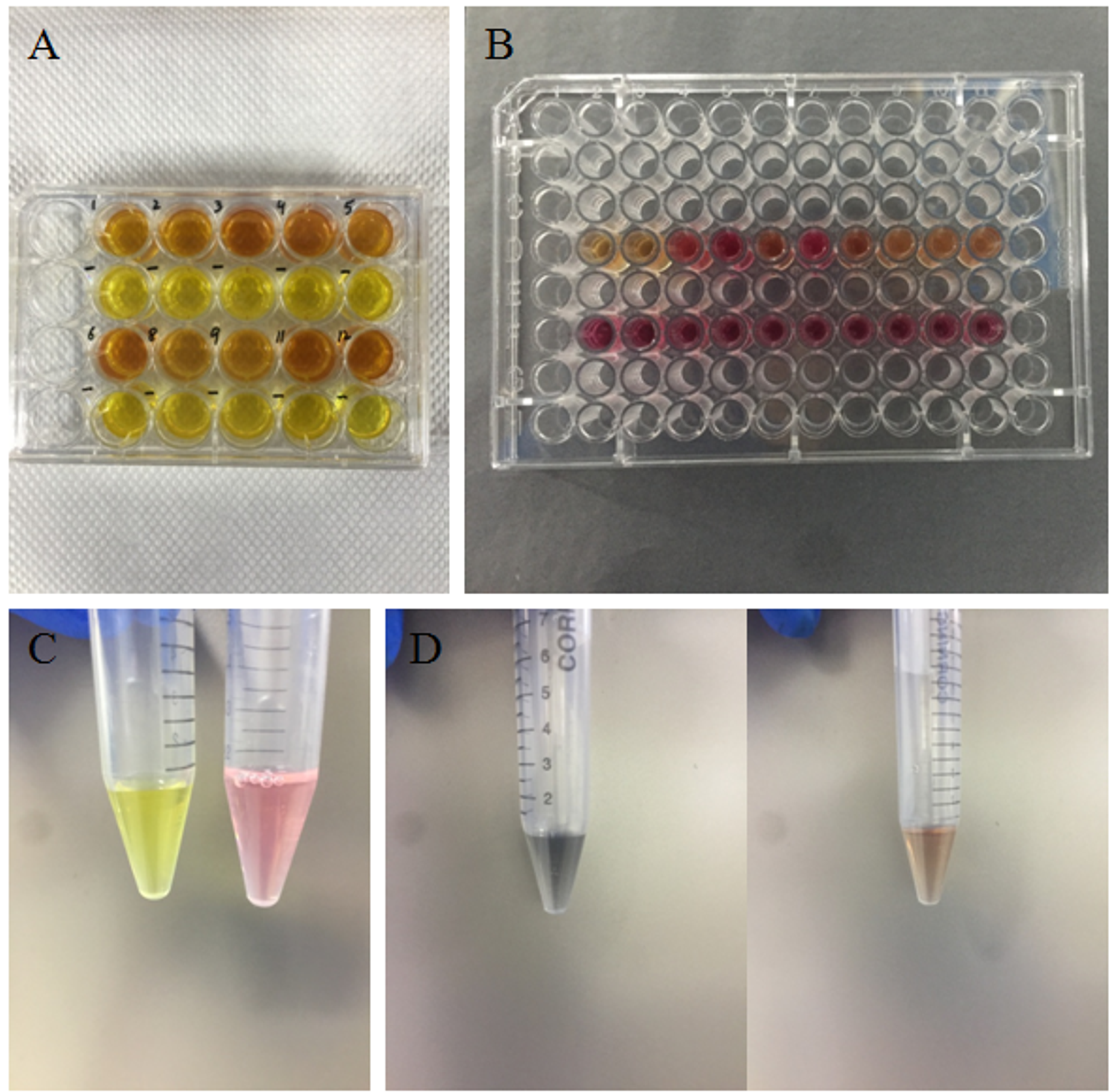

Supplement: S2 Fig — (A) ACC deaminase activity. The upper well with brown coloration showed ACC deaminase detection while the lower well with yellow color was used as negative control. (B) Organic acids production as revealed by a color change to yellow in the upper well, while extreme lower well with pink color was used as negative control. (C) Detection of IAA showing a change of coloration from yellow to pink. (D) Siderophore production was confirmed by a change of color from blue to yellow/orange. (TIF) [file pone.0269640.s002.tif]

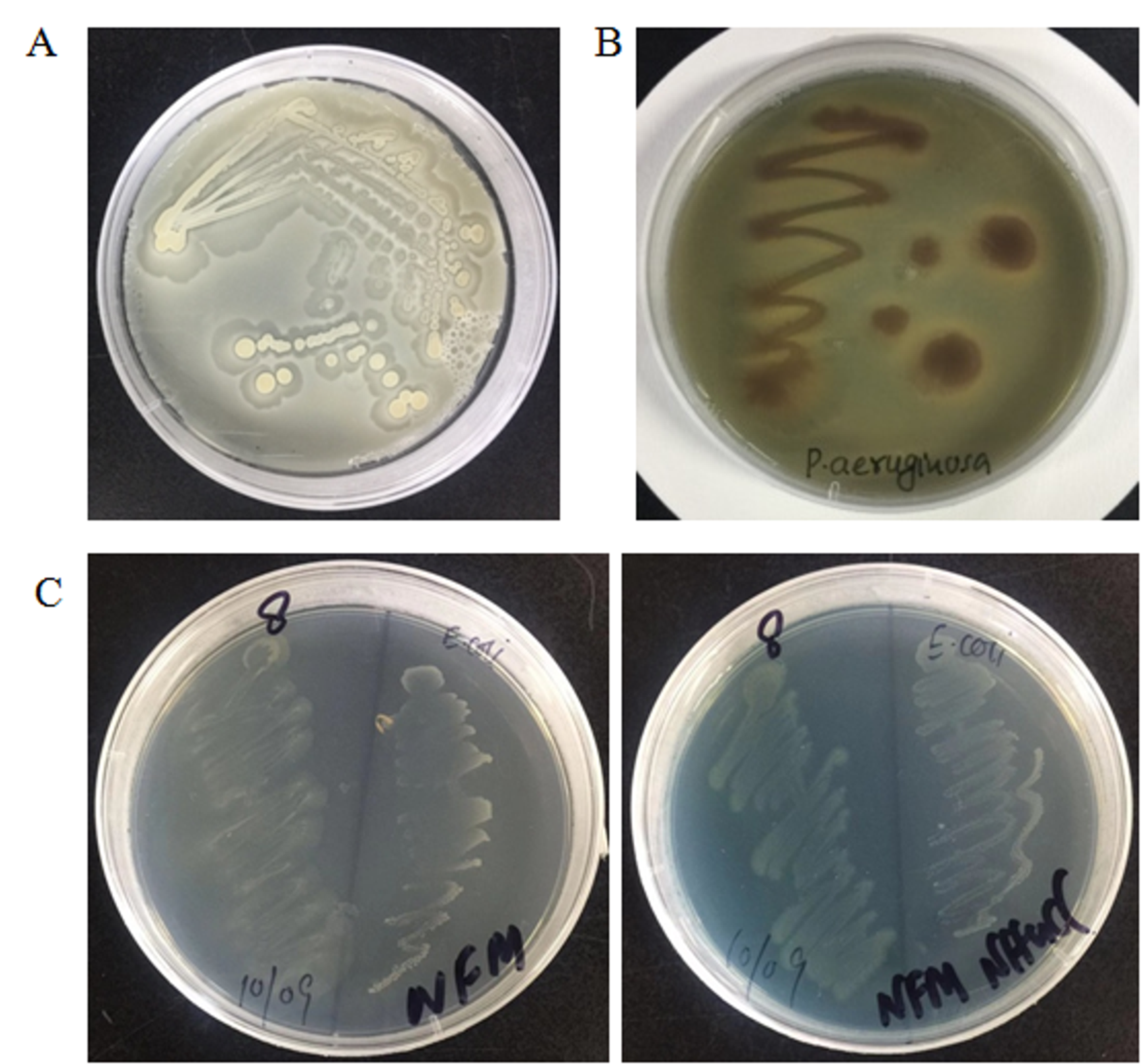

Supplement: S3 Fig — (A) Zone of clearance around colonies of Ld-08 confirming its role in phosphate solubilization. (B) Siderophore release and halo zone formation in CAS agar medium. (C) Nitrogen-fixation assay of Ld-08 strain on nitrogen-deficient malate medium (NFM) and was assessed for growth in reference to non-nitrogen fixing E. coli DH5α on NFM medium, and NFM supplemented with 5 mM NH4Cl. (TIF) [file pone.0269640.s003.tif]
